# Detection of Adult Green Sturgeon Using Environmental DNA Analysis

**DOI:** 10.1371/journal.pone.0153500

**Published:** 2016-04-20

**Authors:** Paul S. Bergman, Gregg Schumer, Scott Blankenship, Elizabeth Campbell

**Affiliations:** 1 Cramer Fish Sciences, West Sacramento, California, United States of America; 2 United States Fish and Wildlife Service, Anadromous Fish Restoration Program, Lodi, Caifornia, United States of America; Chinese Academy of Fishery Sciences, CHINA

## Abstract

Environmental DNA (eDNA) is an emerging sampling method that has been used successfully for detection of rare aquatic species. The Identification of sampling tools that are less stressful for target organisms has become increasingly important for rare and endangered species. A decline in abundance of the Southern Distinct Population Segment (DPS) of North American Green Sturgeon located in California’s Central Valley has led to its listing as Threatened under the Federal Endangered Species Act in 2006. While visual surveys of spawning Green Sturgeon in the Central Valley are effective at monitoring fish densities in concentrated pool habitats, results do not scale well to the watershed level, providing limited spatial and temporal context. Unlike most traditional survey methods, environmental DNA analysis provides a relatively quick, inexpensive tool that could efficiently monitor the presence and distribution of aquatic species. We positively identified Green Sturgeon DNA at two locations of known presence in the Sacramento River, proving that eDNA can be effective for monitoring the presence of adult sturgeon. While further study is needed to understand uncertainties of the sampling method, our study represents the first documented detection of Green Sturgeon eDNA, indicating that eDNA analysis could provide a new tool for monitoring Green Sturgeon distribution in the Central Valley, complimenting traditional on-going survey methods.

## Introduction

Monitoring the distribution of endangered and rare species is critical to assess the status of species recovery and help guide conservation efforts [1]. The low detection probability of rare and imperiled species, coupled with increasing handling restrictions of Federal and State protections, has led to the demand for less stressful sampling methods that at the same time provide greater sensitivity of detection. Environmental DNA (eDNA) is an emerging sampling method that has been used successfully for detection of rare species, and does not require direct contact with the organism [2–3]. Aquatic organisms release DNA into their surrounding environment by leaving behind indicators such as slime, scales, epidermal cells or feces [2]. Environmental DNA has been used to detect the presence of many rare or endangered organisms including Chinook Salmon [1], Asian carp [4], Slackwater Darter [2], giant salamanders [5–6], and various arthropods [7].

The Southern Distinct Population Segment (DPS) of North American Green Sturgeon located in California’s Central Valley were listed as Threatened under the Federal Endangered Species Act in 2006 [8]. The final rule listing indicates that the principle factor for the decline in the DPS is the reduction of spawning to a limited area in the Sacramento River [8]. Terminal dams on the Sacramento, Feather, and Yuba Rivers are believed to have eliminated 100 miles of historical spawning habitat for Southern DPS Green Sturgeon [9]. In addition, an analysis based on the habitat occupied at present versus the habitat available above the dams indicates that Green Sturgeon likely did occupy areas above the dams before dam construction [10].

Although knowledge of Green Sturgeon spawning distribution has increased in recent years with the application of new sampling technologies, knowledge of the spatial extent of spawning and factors driving inter-annual variation in spawning distribution is limited. While abundance of Southern DPS Green Sturgeon is relatively low, adult sturgeon can access hundreds of miles of habitat in the Sacramento River Basin, making spatial distribution monitoring difficult. Southern DPS Green Sturgeon primarily spawn in cool sections of the upper mainstem Sacramento River in deep pools containing gravel, cobble, or boulder substrate from April through early July [11– 12]. Since 2010, Dual Frequency Identification Sonar (DIDSON) surveys of spawning sites of Green Sturgeon have been conducted along the Sacramento River and have identified numerous spawning areas across a 75 mile stretch of the river (pers. comm. with Ethan Mora, UC Davis). In addition, studies have identified spawning locations in the Feather River, a tributary of the Sacramento River, and indicate variability in tributary spawning related to annual flow regime, with more adult observations following high flow events [13]. Despite greater knowledge of locations of Green Sturgeon spawning concentrations from DIDSON surveys, little is still known about habitat usage in other Sacramento River tributaries and how extent of spawning varies across years.

The relatively quick and inexpensive nature of eDNA analysis [3] could make it useful for monitoring Green Sturgeon distribution due to the large spatial extent of Green Sturgeon spawning in the Central Valley. In addition, eDNA detection methods have been shown to be more sensitive than traditional sampling methods at determining presence of rare species [1]. The high detection sensitivity of eDNA analysis could provide information on distribution of Green Sturgeon spawning in even the most underutilized habitats, helping target specific habitats for full ecological surveys.

However, like any new sampling tool, studies first need to be conducted to examine the effectiveness of using eDNA analysis to monitor Green Sturgeon prior to implementing large-scale monitoring efforts. Because detection efficiency has been shown to vary by species [14], the first step is to determine if Green Sturgeon eDNA can be detected in locations of known presence. If detection is successful, future studies can examine how detection probability varies under differing environmental conditions, fish densities, and distances from known sturgeon, in order to help inform an effective sampling design for monitoring Green Sturgeon distribution. Therefore, the objective of this study was to determine if eDNA analysis could be used to positively identify adult Green Sturgeon in locations of known presence in the Sacramento River.

## Methods

**Fig 1:**
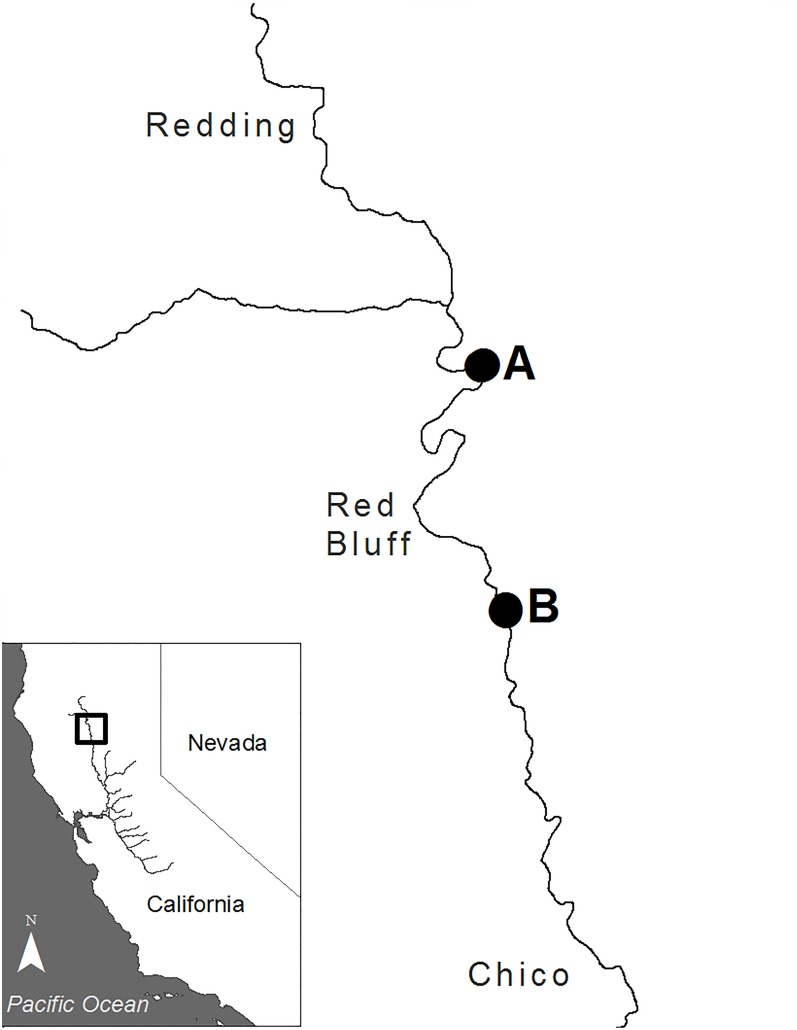
Environmental DNA sampling locations on the Sacramento River, CA.

Water sampling was conducted at two locations of known presence of adult Green Sturgeon in the Sacramento River near the city of Red Bluff, CA (Fig 1). Both locations occur in public bodies of water with no permission for sampling required. Sampling at each location occurred within 24 hours of confirmed presence of multiple Green Sturgeon identified during roving DIDSON surveys conducted by University of California- Davis (pers. comm. with Ethan Mora, UC Davis). Both sampling locations are deep pool habitats with average depths of approximately 5 meters. Both sites have been observed to perennially hold Green Sturgeon throughout the spawning period (pers. comm. with Ethan Mora, UC Davis), and Green Sturgeon eggs have been sampled from each location during multiple field seasons [12,15,16].

Water samples were collected at 100 meter intervals at each sampling location using a Garmin Oregon (Garmin Ltd.) on 11 June, 2015 at location B and 30 June, 2015 at location A. Water samples were collected at the site of known Green Sturgeon presence (Sample A2 for location A and Sample B2 for location B), 100 m upstream, and at 100 m and 200 m downstream (Table 1). To provide adequate sampling coverage across the river channel, parallel samples were taken at stream right and left at the 100 m and 200 m downstream distance for location B only, for a total of 4 samples collected at location A and 6 samples at location B.

**Table 1:** Results of qPCR analysis for Green Sturgeon DNA (“+” = positive, “−” = negative) at locations A and B in the Sacramento River, quantification cycle (Cq) for each technical replicate of a positive test for Green Sturgeon DNA, and average Cq across replicates.

Sample	Results	Cq 1	Cq 2	Cq 3	Avg. Cq
**A1**	+	38	ND*	ND	38
**A2**	+	34	ND	35	35
**A3**	+	37	ND	ND	37
**A4**	+	35	ND	35	35
**B1**	+	34	35	37	35
**B2**	+	37	ND	ND	37
**B3**	+	36	35	36	36
**B4**	+	35	35	ND	35
**B5**	+	35	35	35	35
**B6**	+	36	36	ND	36
**Negative Field Control 1**	−	N/A	N/A	N/A	N/A
**Negative Field Control 2**	−	N/A	N/A	N/A	N/A
**Extraction Controls**	−	N/A	N/A	N/A	N/A
**No Template Controls**	−	N/A	N/A	N/A	N/A
**Positive Control 1**	+	19	19	19	19
**Positive Control 2**	+	19	19	18	19

For each sampling event we directly filtered two L of water from the Sacramento River at a an approximate depth of ≤ 6 inches below the surface using sterile Saint Gobain XL-60 silicon tubing (Tygon^®^; internal diameter 6.3mm), and a portable Masterflex^®^ L/S Easy-Load II peristaltic pump(Cole-Parmer^®^) powered by a cordless hand drill. Water samples were filtered through a Millipore Sterivex^™^-GP 0.22μm sterile filter unit (EMD Millipore). No water was transported or stored during sampling nor was any water transported between sampling sites; instead all filtration occurred directly on the boat at each site. Sample filtrate was captured and measured in graduated flasks to verify the volume of each sample. Filtered water was then poured over the side of the boat after completion of sampling at each site. To eliminate cross contamination between sites due to equipment, all tubing was considered single use, was used for 1 sample only and immediately disposed of after each use into a sealed trash bag on board. All filters are likewise considered single use. Filters come from the manufacturer individually wrapped in order to maintain sterility and were opened immediately prior to use. After filtration, the cylindrical filters were capped at each end, labelled with location ID, placed into a sterile secondary container, sealed, and immediately placed on ice. All filters were kept on ice in a cooler for the duration of the sampling event until they were transferred to a -20°C laboratory freezer. The filters were stored within individually sealed secondary containers at -20°C until DNA extraction. To ensure that field equipment was free of contaminating DNA field controls were taken for each sample day. Each field control consisted of Sterivex^™^ filtered ultra-pure water pumped using the portable peristaltic pump used to take all samples and a new length of sterile silicon tubing. The field controls were then processed for the presence of Green Sturgeon DNA in parallel with all samples.

We conducted DNA extractions using PowerWater^®^ Sterivex^™^ DNA Isolation Kit (Mo Bio Laboratories, Inc.) following the manufacturer’s recommended guidelines. We conducted DNA extractions in a laboratory that had never extracted Green Sturgeon DNA prior to this study. A DNA extraction negative control was processed in parallel to ensure sample integrity throughout extraction procedure. The DNA extraction control consisted of Sterivex^™^ filtered ultrapure water only. DNA extraction controls were processed using the same equipment utilized to extract DNA from all samples.

Each sample and all controls were analyzed in triplicate for the presence of the Green Sturgeon COI mitochondrial gene using a qPCR primer and probe set as previously described by Brandl et al. [17](Accession number KF558288, Fwd-AGGGAAAAAATGGTTAGGTCTACAGA Rev-CCCCACTGGCGGGAAA Probe-CTCCCGCATGGGCTA). Brandl et al also demonstrated the Green Sturgeon primer probe set to be species specific thus does not amplify the DNA of other closely related co-existing species. Each qPCR replicate consisted of a 5 ul reaction volume. Each 5 ul qPCR reaction was composed of 1x Applied Biosystems TaqMan Universal PCR Master Mix, No AmpErase UNG (Applied Biosystems ^™^), 900nm final primer concentration, 60nm final probe concentration, and 1 ul DNA template. Thermocycling was performed using a Bio-Rad CFX 96 Real time System (Bio-ad Laboratories, Inc.) with the following profile: 10 minutes at 95°C, 40 cycles of 15 second denaturation at 95°C and 1 minute annealing-extension at 60°C. Six template control (NTC) reactions were run on the plate with the samples template controls consisted of 1ul of ultrapure water replacing DNA template within reaction volume. Three positive control reactions consisting of 20ng/ul Green Sturgeon genomic DNA template were also tested in parallel to ensure consistent PCR performance. All PCR master mixes were made inside a UV PCR enclosed workstation. DNA template was added to master mix outside of the UV PCR workstation on a dedicated PCR set up workbench. All PCR reactions were conducted on instruments located outside of the main lab in a separate portion of the building. Results of the qPCR reactions were analyzed using BioRad CFX manager v3.1 (Bio-Rad Laboratories, Inc.). A sample was considered positive for the presence of Green Sturgeon DNA if any one of the three replicates showed logarithmic amplification within 40 cycles.

## Results

Green Sturgeon eDNA were detected in all 10 samples from the Antelope Creek and Massacre Flat sampling locations (Table 1). All control samples were negative for target eDNA. The average quantification cycle (Cq) when Green Sturgeon DNA was positively identified varied from 34–37 (mean = 36) across the 10 samples (Table 1).

## Discussion

We positively identified Green Sturgeon DNA at two locations of known presence in the Sacramento River, proving that eDNA can be effective for monitoring the presence of adult sturgeon. Environmental DNA analysis has also been effective at identifying presence of other fish species in lotic habitats, such as Chinook salmon [1], Brook Trout [18], Slackwater Darter [2], and invasive Asian carp [4]. Although our findings are preliminary, our results concurred with traditional visual survey methods, indicating that eDNA may provide a new survey tool for monitoring Green Sturgeon distribution.

Environmental DNA analysis provides a more cost-effective and less-invasive sampling method compared to traditional survey techniques [3]. Because field sampling only requires collecting a water sample, eDNA analysis can have considerable time and cost benefits over traditional sampling, allowing for greater spatial distribution of effort, which is particularly important for adult Green Sturgeon that have access to hundreds of miles of habitat in the Sacramento River Basin. In a study of invasive Asian carp in Chicago, Illinois, it took 93 days of person effort to detect one Silver Carp by electrofishing at a site, whereas eDNA analysis required only 0.174 days person effort to achieve a positive detection [4]. In addition to being cost-effective, eDNA analysis has no risk of harming species under study [4]. The Identification of sampling tools that are less stressful for target organisms has become increasingly important for rare and endangered species, such as Green Sturgeon.

Although our initial findings show promise for applying eDNA analysis for monitoring Green Sturgeon, greater study is needed to examine factors influencing variability in eDNA detection rates. An understanding of the sensitivity of eDNA detection rates downstream in running waters is critical to make eDNA analysis a satisfactory survey method [14]. Similar to our findings, a study found Brook Trout eDNA detectable up to 239.5 meters downstream [18]. Deiner and Altermatt [19] were able to detect DNA of daphnia and mussels nearly 20 km downstream of their source. Although studies are needed to examine the maximum detection distance of Green Sturgeon DNA, the extreme sensitivity of eDNA detection observed in other studies may limit its utility to only delineating the upstream extent of Green Sturgeon habitat occupancy. In addition, an understanding of how eDNA dilution varies with hydrologic conditions is also an important consideration for interpreting eDNA results [14]. For example, Jane et al. [18] found that detection rates of Brook Trout DNA were lower under high flow conditions at all distances away from the target organisms. Experimental studies specific to Green Sturgeon are needed in order to scale up results from local water samples.

While studies have shown the utility of using eDNA for presence/absence, much is still unknown about estimating biomass, making more traditional survey methods still important for estimating population size [3]. Studies have found quantity of eDNA to be correlated with species density for Common Carp [20], bigheaded carps [21], and Rocky Mountain Tailed Frogs and Idaho Giant Salamanders [22]. While these studies are promising, there is limited knowledge of how conditions such as fish behavior (DNA shedding rates), water chemistry, UV-B exposure, and water temperature affect eDNA concentration [3]. For eDNA analysis to be a useful tool for estimating Green Sturgeon biomass, experimental studies are needed to understand how these factors influence eDNA concentration and resulting biomass estimates.

Environmental DNA analysis could provide a new tool for monitoring Green Sturgeon distribution in the Central Valley, complimenting traditional on-going survey methods. Traditional survey methods have identified perennial Green Sturgeon spawning locations in the mainstem of the Sacramento River [12,15,16], and surveys in the Feather River [13] and Yuba River [23] indicate that Green Sturgeon opportunistically spawn in some Sacramento River tributaries during high flow conditions. Currently, the primary tool for monitoring Green Sturgeon spawning distribution in the Sacramento River Basin is field-intensive roving DIDSON surveys. While visual surveys are effective at monitoring fish densities in concentrated pool habitats, results do not scale well to the watershed level, providing limited spatial and temporal context. In contrast, environmental DNA analysis provides a relatively quick, inexpensive tool that could efficiently monitor the presence and distribution of Green Sturgeon throughout the entire Sacramento River Basin and help explore factors driving fish presence. Findings of large-scale eDNA monitoring could then be used to target specific water bodies for conservation efforts or for full ecological surveys [3].

Our study represents the first documented detection of Green Sturgeon eDNA. While additional study is needed to refine the sampling technique, eDNA analysis provides a non-invasive, low-cost alternative to traditional survey techniques that shows promise for detecting Green Sturgeon presence. Better knowledge of the spatial extent of Green Sturgeon spawning could help identify previously unknown spawning habitats and discover factors influencing habitat usage, helping direct future conservation efforts.

## References

[1] Laramie MB, Pilliod DS, Goldberg CS. Characterizing the distribution of an endangered salmonid using environmental DNA analysis. Biological Conservation. 2015;183:29–37.

[2] Janosik AM, Johnston CE. Environmental DNA as an effective tool for detection of imperiled fishes. Environmental Biology of Fishes. 2015;98:1889–1893.

[3] Rees HC, Maddison BC, Middleditch DJ, Patmore JRM, Gough KC. The detection of aquatic animal species using environmental DNA—a review of eDNA as a survey tool in ecology. Journal of Applied Ecology 2014;51:1450–1459.

[4] Jerde CL, Mahon AR, Chadderton WL, Lodge DM. “Sight-unseen” detection of rare aquatic species using environmental DNA. Conservation of Letters. 2011;4:150–157.

[5] Fukumoto S, Ushimaru A, Minamoto T. A basin-scale application of environmental DNA assessment for rare endemic species and closely related exotic species in rivers: a case study of giant salamanders in Japan. Journal of Applied Ecology. 2015;52:358–365.

[6] Goldberg CS, Pilliod DS, Arkle RS, Waits LP. Molecular detection of vertebrates in stream water: a demonstration using rocky mountain tailed frogs and Idaho giant salamanders. PLOS ONE. 2011; 6(7): e22746 10.1371/journal.pone.0022746 21818382 PMC3144250

[7] Thomsen PF, Kielgast J, Ivesen LL, Wiuf C, Rasmussen M, Golbert MTP, et al Monitoring endangered freshwater biodiversity using environmental DNA. Molecular Ecology. 2012;21:2565–2573. 10.1111/j.1365-294X.2011.05418.x 22151771

[8] National Marine Fisheries Service (NMFS). Endangered and threatened species: endangered and threatened wildlife and plants: threatened status for Southern Distinct Population Segment of North American Green Sturgeon. Federal Register. 2006;71:17757–17766.

[9] National Marine Fisheries Service (NMFS). Green sturgeon (Acipenser medirostris) status review update. Santa Cruz (CA): Biological Review Team, Southwest Fisheries Science Center; 2005 2 Available: http://www.nmfs.noaa.gov/pr/pdfs/statusreviews/greensturgeon_update.pdf

[10] Mora EA, Lindley ST, Erickson DL, Klimley AP. Do impassable dams and flow regulation constrain the distribution of green sturgeon in the Sacramento River, California. Journal of Applied Ichthyology. 2009;25:39–47.

[11] National Marine Fisheries Service (NMFS). Southern Distinct Population Segment of the North American Green Sturgeon: 5-year review: summary and evaluation. Long Beach (CA):National Marine Fishereis Service; 2015 8 Available: http://www.westcoast.fisheries.noaa.gov/publications/protected_species/other/green_sturgeon/8.25.2015_southern_dps_green_sturgeon_5_year_review_2015.pdf

[12] Poytress WR, Gruber JJ, Praetorius CE, Van Eenennaam JP. 2012 Upper Sacramento River green sturgeon spawning habitat and young of the year migration surveys. Redd Bluff (CA): U.S. Fish and Wildlife Service; 2013 Sep. Annual Report. Sponsored by U.S. Bureau of Reclamation.

[13] Seesholtz A, Manuel M, Van Eenennaam J. First documented spawning and associated habitat conditions for green sturgeon in the Feather River, California. Environmental Biology of Fishes. 2014;98:905–912.

[14] Roussel J-M, Paillisson J-M, Treguier A, Petit E. The downside of eDNA as a survey tool in water bodies. Journal of Applied Ecology. 2015;52:823–826.

[15] Poytress WR, Gruber JJ, Trachtenbarg A, Van Eenennaam JP. 2009 upper Sacramento River green sturgeon spawning habitat and larval migration surveys. Redd Bluff (CA): U.S. Fish and Wildlife Service; 2010 Jul. Annual Report. Sponsored by U.S. Bureau of Reclamation.

[16] Poytress WR, Gruber JJ, Trachtenbarg A, Van Eenennaam JP. 2008 upper Sacramento River green sturgeon spawning habitat and larval migration surveys. Redd Bluff (CA): U.S. Fish and Wildlife Service; 2009 Mar. Annual Report. Sponsored by U.S. Bureau of Reclamation.

[17] Brandl S, Schumer G, Schreier BM, Conrad JL, May B, Baerwald MR. Ten real-time PCR assays for detection of fish predation at the community level in the San Francisco Estuary-Delta. Molecular Ecology Resources. 2015;15:278–284. 10.1111/1755-0998.12305 25042458

[18] Jane SF, Wilcox TM, Mckelvey KS, Young MK, Schwartz MK, Lowe WH, et al Distance, flow and PCR inhibition: eDNA dynamics in two headwater streams. Molecular Ecology Resources. 2015;15:216–227. 10.1111/1755-0998.12285 24890199

[19] Deiner K, Altermatt F. Transport distance of invertebrate environmental DNA in a natural river. PLOS ONE. 2014; 9(2): e88786 10.1371/journal.pone.0088786 24523940 PMC3921251

[20] Takahara T, Minamoto T, Yamanaka H, Doi H, Kawabata ZI. Estimation of fish biomass using environmental DNA. PLOS ONE. 2012; 7(4): e35868 10.1371/journal.pone.0035868 22563411 PMC3338542

[21] Klymus KE, Richter CA, Chapman DC, Paukert C. Quantification of eDNA shedding rates from invasive bighead carp *Hypophthalmichthys nobilis* and silver carp *Hypophthalmichthys molitrix*. Biological Conservation. 2015;183:77–84.

[22] Pilliod DS, Goldberg CS, Arkle RS, Waits LP. Estimating occupancy and abundance of stream amphibians using environmental DNA from filtered water samples. Canadian Journal of Fisheries and Aquatic Sciences. 2013;70:1123–1130.

[23] Bergman PS, Merz J, Rook B. Memo: green sturgeon observations at Daguerre Point Dam, Yuba River, CA. Auburn (CA): Cramer Fish Sciences; 2011 6. Grant No.: 813329G011. Sponsored by U.S. Fish and Wildlife Service.

